# Ethical challenges in treatment-goal transitions in invasively ventilated ALS: a case-based topical review

**DOI:** 10.1186/s42466-026-00522-3

**Published:** 2026-07-30

**Authors:** Sarah K. Bublitz, Benedikt Becker, Antonia F. Demleitner, Petra Dietz-Laukemann, Paul Lingor, Benno Littger, Stefan Lorenzl, Berend Feddersen

**Affiliations:** 1 Department of Neurology, Agatharied Hospital, Hausham, Germany; 2 Institute of Palliative Care, Paracelsus Medical University, Salzburg, Austria; 3 Department of Neurology, TUM School of Medicine and Health, TUM University Hospital, Technical University of Munich, Munich, Germany; 4 Department of Palliative Medicine, LMU University Hospital, LMU Medizin, Ludwig Maximilians-Universität München, Munich, Germany; 5 Archdiocese Munich and Freising, Munich, Germany

**Keywords:** Amyotrophic Lateral Sclerosis, Tracheostomy-invasive ventilation, Communication, Advance Care Planning, Decisional capacity, Moral distress

## Abstract

**Introduction:**

In advanced amyotrophic lateral sclerosis (ALS), eye movements often represent the last channel for intentional communication. While oculomotor function has traditionally been considered relatively preserved, emerging evidence indicates progressive impairment in long-term survivors on tracheostomy-invasive ventilation (TIV). As a result, eye-based communication may become increasingly unreliable before complete loss, challenging clinical decision-making and advance care planning (ACP).

**Methods:**

We conducted a case-based topical review integrating clinical observation and literature to examine the trajectory of oculomotor decline, its impact on communication, and implications for treatment decisions. Three patients with ALS receiving TIV in a home-care setting illustrate key clinical and ethical challenges.

**Results:**

Across cases and literature, oculomotor decline followed a gradual trajectory from effective eye-tracking communication to a complete locked-in syndrome. We identify a transitional phase of *communicative ambiguity*, in which residual ocular signals persist but can no longer be reliably attributed to intentional, patient-controlled communication. This phase is characterized by increasing inconsistency of signals and a divergence between observable responses and their interpretive certainty, creating uncertainty in assessing patient preferences.

**Implications:**

Communicative ambiguity represents a clinically underrecognized but critical threshold in advanced ALS, marking the transition from direct patient autonomy to interpretative and surrogate-based decision-making. Failure to recognize this phase risks misinterpretation of patient intent and may undermine goal-concordant care. Timely and iterative ACP, initiated before communication becomes unreliable, is essential. We further propose four clinical pathways for treatment goal conversations, highlighting their differing implications for timing, symptom burden, and ethical decision-making.

## Background

Oculomotor function has traditionally been regarded as relatively preserved in Amyotrophic Lateral Sclerosis (ALS). However, emerging evidence challenges this assumption and indicates that oculomotor dysfunction may occur in patients with prolonged survival [1, 2], especially when receiving tracheostomy-invasive ventilation (TIV). In non-ventilated ALS, death typically occurs in the context of hypercapnic respiratory failure, frequently accompanied by reduced consciousness due to carbon dioxide narcosis [3]. While invasive ventilation can significantly prolong survival [4, 5], it does not halt neurodegeneration [6].

Complete loss of communication has been recognized as a critical stage in ALS using TIV [7, 8], and a reason for withdrawal of ventilation [9–11]. In a German cohort of ALS patients who had long-term ventilation withdrawn, 41% of patients showed complete or partial ophthalmoplegia [12].

Reported oculomotor abnormalities in ALS include slowing of saccades, impaired smooth pursuit, and deficits in inhibitory control, reflecting involvement of both reflexive and executive eye movement systems in parallel to neuropathological stagings of the disease [13–16]. These changes occur in parallel with broader neurodegenerative processes and may be associated with cognitive impairment and executive dysfunction [17, 18], as well as bulbar onset [17, 19]. Rapid disease progression seems to indicate future communication impairment after the use of TIV [20], and some correlations have been found between eye movement deficits and extra-motor clinical signs [21]. Eye-tracking–based studies provide insight into the functional consequences of these deficits. Early alterations, such as increased error rates in antisaccade tasks, indicate reduced inhibitory control and impaired voluntary gaze regulation. With disease progression, patients develop prolonged saccadic latencies, hypometric and dysmetric eye movements, and reduced smooth pursuit gain. In advanced stages, these impairments converge toward a global oculomotor dysfunction, which may ultimately result in a completely locked-in state with total ophthalmoplegia [16, 22]. While a completely locked-in state in ALS is typically considered an extreme stage of the disease that develops over several years [23–25], reports indicate that such states can also emerge over shorter timeframes [20, 22].

For patients with advanced ALS, eye movements often constitute the last available channel for intentional communication. Eye-tracking communication systems (ETCS) depend on stable gaze fixation, accurate target selection, and reproducible voluntary control. In progressive oculomotor impairment, slowed initiation of voluntary saccades reduces communication speed, while hypometric or dysmetric saccades impair target selection and calibration. Impaired suppression of reflexive eye movements increases unintended selections, and smooth pursuit deficits further compromise tracking-based interfaces. Beyond central oculomotor deficits, peripheral ocular factors can significantly affect ETCS usability. Prolonged visual attention reduces spontaneous blink rate, impairing tear film stability and causing ocular surface exposure and discomfort. These effects may be exacerbated by medications such as anticholinergics and psychotropic agents [26]. Additionally, tear production appears to be reduced in ALS [27].

ETCS reliability declines not only quantitatively, through slower and less efficient interaction, but also qualitatively, through increasing ambiguity of gaze signals. Patients may still produce gaze fixations, minimal eyelid movements, or isolated directional eye shifts. However, the distinction between intentional, fatigue-related, and reflexive movements becomes progressively difficult. We define this state as *communicative ambiguity*, referring to a condition in which ocular signals can no longer be reliably attributed to intentional, reproducible, and patient-controlled communication.

ALS is associated with mild to moderate cognitive impairment in 35–45% during the course of the disease with 15% of patients eventually meeting criteria for the diagnosis of Frontotemporal Dementia (FTD) itself [28, 29]. Importantly, features of FTD may lead to cognitive and behavioral changes that compromise decision-making capacity [30] and further complicate the interpretation of patients’ preferences at that stage.

The phase of communicative ambiguity represents a clinically critical transition period: a time window in which patients retain the ability to communicate but are at imminent risk of losing this capacity. Within this window, it is essential to clarify whether and when the therapeutic goal should shift from life-sustaining treatment to a primarily palliative, symptom-oriented approach, with or without withdrawal of life-sustaining therapies like nutrition or ventilation. If this transition is not addressed in time, subsequent loss of communication precludes confirmation of the patient’s will, and decisions must be made under substantial uncertainty by surrogates. Family members and legal representatives may experience this situation as being forced to make life-and-death decisions on behalf of the patient. Up to 30% of relatives involved in such decision-making processes develop symptoms consistent with post-traumatic stress disorder (PTSD) [31, 32].

A stage of *communicative ambiguity* is also seen in numerous other clinical conditions. In ALS, however, it has specific implications for patients, their families, and healthcare providers, as the combination with TIV can force particularly burdensome treatment decisions. Advance care planning (ACP) provides a structured framework to address this challenge. ACP enables patients, based on their individual values and beliefs, to define and document their preferences regarding future medical treatment, including conditions under which life-sustaining interventions should be continued or limited [33, 34].

Neurologists are often no longer regularly involved in the care of ALS patients at advanced stages [35], as patients are unable to attend outpatient clinics, and home visits by neurologists are scarce. Nevertheless, neurologists play a key role in identifying this critical time window and initiating timely, structured discussions. In practice, care is frequently provided by specialized palliative home care teams, making close coordination and collaboration between disciplines essential. In many healthcare systems, these patients are not admitted to palliative care units lacking invasive ventilation capabilities, nor to intensive care units for the purpose of treatment withdrawal, making home-based care the primary setting for end-of-life management.

While complete loss of communication has been discussed in the context of end-of-life decision-making, the preceding period, characterized by increasingly unreliable and ambiguous signals, remains poorly understood and lacks clinical and ethical guidance. In this phase, fundamental assumptions underlying patient autonomy and informed decision-making are challenged, as it becomes uncertain whether observed responses can still be interpreted as valid expressions of will. This raises key questions: At what point should previously expressed preferences take precedence over ambiguous signals? How should clinicians and surrogate decision-makers navigate treatment decisions under conditions of profound uncertainty?

To address these questions, this topical review aims to synthesize evidence on clinical characteristics and conceptualization of communicative ambiguity, and the ethical and practical implications for decision-making and advance care planning in patients with ALS receiving TIV.

## Methods

We conducted a case-based topical review focusing on the intersection of progressive oculomotor decline in advanced ALS, loss of communication, and implications for treatment goal transitions. Literature was identified through targeted, non-systematic searches in PubMed and reference lists of relevant articles using combinations of terms including A*myotrophic Lateral Sclerosis*, *tracheostomy-invasive ventilation*, *ophthalmoplegia*, *eye movement*, *communication loss*, *eye-tracking communication*, *advance care planning*, *goals of care*, *withdrawal of ventilation*, and *end-of-life care*. Selection was guided by clinical relevance and conceptual contribution, with particular emphasis on studies addressing advanced ALS under invasive ventilation, communication via eye-tracking systems, ACP, and end-of-life decision-making.

To illustrate the clinical and ethical challenges, three exemplary cases of patients with ALS receiving tracheostomy-invasive ventilation within a specialized homecare program in Bavaria, Germany, are presented [36]. Ethics approval was obtained from the ethics commission of the Ludwig-Maximilians-University of Munich (project no 21–0991), and all patients or their legal representatives provided informed consent.

### Case illustrations

#### Case illustration 1

A 64-year-old woman with spinal-onset ALS initially refused invasive ventilation but later consented to tracheostomy and PEG due to acute respiratory decline. She established an advance directive specifying that mechanical ventilation should be discontinued upon loss of eye-movement communication.

Sixteen months post-tracheostomy (and 30 months after disease onset), oculomotor function deteriorated, rendering her eye-tracking computer system (ETCS) unusable. At 25 months post tracheostomy, only residual horizontal gaze and inconsistent eyelid signals remained, leading to “profound uncertainty” regarding her decisional capacity. Based on her advance directive, the palliative team and family agreed to omit antibiotics for future infections. However, this triggered moral distress among nursing staff, who interpreted subtle ocular flickering as a “will to live.” Tensions further escalated between siblings regarding the prolongation of suffering due to pain during a subsequent keratitis. The patient died after a prolonged phase of minimal, unreliable communication, rather suddenly 53 months after diagnosis (40 months post-tracheostomy).

#### Case illustration 2

A 44-year-old man underwent emergency tracheostomy one month after ALS diagnosis. Communication was maintained via ETCS and he initially requested full intensive care to see his young daughter grow up, but stated he would reconsider life-sustaining treatment if ETCS communication failed. Attempts to clarify treatment limits caused significant emotional distress, and the patient rejected further discussion.

As oculomotor control became inconsistent, a specialized palliative care team was involved to support ACP and address psychosocial and existential concerns. The patient articulated a profound attachment to life, closely linked to his identity as a father. Life-prolonging treatment during medical crises was acceptable, particularly if it enabled him to remain at home, as he feared hospitalization might prevent a return home and compromise his wish to die there. He struggled to define a clear line at which the therapeutic goal should be changed, eventually proposing a six-month trial period of life in a near-locked-in state (LIS) before life-sustaining treatments should be discontinued.

By month 25 after diagnosis, vertical eye movements had ceased, and only horizontal gaze was inconsistently possible, signalling a transition to a completely locked-in state (CLIS). Despite previous hesitations, he requested ICU admission for pneumonia at month 29 via reproducible horizontal eye movements. The subsequent ten-day period of intensive care was later described by his wife as highly stressful. Two months later, with all reliable communication lost, an interdisciplinary ethics committee concluded that continued ventilation no longer aligned with his previously expressed values. His wife interpreted his prior statements that he would not have wished for continued ventilation beyond a certain, albeit not precisely defined, point in time.

Parallel to the patient’s neurological deterioration, the psychosocial burden on the family intensified. His wife increasingly withdrew emotionally, focusing on professional responsibilities and childcare, while their daughter expressed concern that her father might be suffering.

Following this consensus, antibiotics, nutrition, and hydration were withheld during a recurrent pneumonia. A distant relative raised serious accusations, despite repeated efforts by the palliative care team to provide information and explain the situation, claiming that the team was allowing the patient to starve and dehydrate. He died 17 days later, 34 months after diagnosis and 33 months after tracheostomy.

#### Case illustration 3

A 56-year-old male with *SOD1*-associated ALS was treated with tofersen, which served as a significant psychological source of hope. Following tracheostomy 18 months after diagnosis, he remained stable for months until repeated septic complications and ETCS failure due to fatigue and declining blink frequency led to diminished quality of life. He emphasized the central importance of communication, stating that losing the ability to express needs and wishes would represent a critical threshold. At the same time, he continued to report a meaningful quality of life in the presence of his family. 36 months after diagnosis, as hypercapnic episodes increased, ACP discussions with the specialist palliative care team resulted in a decision to avoid further hospitalizations and to manage crises at home. In the following weeks, he expressed loss of hope and began considering withdrawal of ventilation even before complete loss of communication. As periods of reduced vigilance increased and periods of communication using blinking to signal yes/no were becoming scarce, an ethical case discussion concluded that a threshold had been reached at which the patient no longer considered his condition worth living. After the case discussion, he was able to confirm, via blinking, his wish not to continue life-sustaining treatment towards his adult children; this was one of the last instances of successful communication.

He was started on palliative sedation, artificial nutrition and hydration were discontinued, and ventilation was withdrawn 41 months after diagnosis and 23 months after tracheostomy, in the presence of his family.

Subsequently, a conflict emerged with the intensive home care service, whose representatives, although present during the ethics case consultation, later questioned whether the decision had adequately reflected the patient’s will. This illustrates the ethical tensions and retrospective uncertainties that may persist even after a consensus-oriented decision-making process.

### Synthesis of literature

#### Impact of oculomotor decline on decision-making and advance care planning in ALS

Advance care planning (ACP) is a central component of multidisciplinary care in ALS and is recommended in neurological guidelines, emphasizing the need for discussions about prognosis, treatment options, and patient preferences [37, 38]. Given the progressive nature and the risk of losing communication capacity, timely ACP enables patients to articulate their values and goals of care, including preferences regarding life-sustaining interventions such as gastrostomy and ventilation. Early integration of neuropalliative care has been associated with better care coordination and service delivery and a higher rate of Advance Medical Directive completion [39]. Despite this, ACP implementation in ALS remains inconsistent. A recent systematic review demonstrated substantial international variability in the prevalence and content of advance directives [40], while a German survey found that loss of communication ability was explicitly cited in 48% of directives as a condition for discontinuing life-prolonging treatment, yet documented medical counselling was present in only 44% of cases [41]. These findings suggest that while patients recognize the importance of anticipating future loss of autonomy, structured and supported ACP is not consistently achieved.

Decision-making in ALS is shaped by psychological, relational, cultural, spiritual and temporal factors and interaction between disease progression, emotional coping, relational dynamics, and temporally extended autonomy. Qualitative studies show that patients and families experience ACP as an ongoing process of coping with progressive loss while maintaining hope and a sense of control, frequently adopting a present-focused orientation [42]. While professionals often initiated discussions about medical end-of-life decisions, patients and families emphasized relational responsibilities, emotional readiness, and quality of life, highlighting the multidimensional nature of ACP beyond pure medical decision-making. Decisions in ALS are not seen as discrete events but evolve over time, and may be deliberately postponed as a way to preserve autonomy and identity [43] or be revoked as an effect of the “disability paradox” [44]. While such postponement can represent an expression of self-determination, it may also entail risks, as disease progression, and particularly oculomotor decline, may limit both the feasibility of interventions and the patient’s ability to communicate preferences. Opportunities to express values and guide future care may be irreversibly lost. Recent evidence confirms that delayed timing of these discussions is a recurring clinical reality [45], which increases the risk that critical decisions are addressed after reliable communication has already been compromised.

#### The “Gray Zone” of communicative ambiguity

The three cases illustrate that progressive failure of ETCS and eventual ophthalmoplegia introduce distinct ethical and clinical challenges in advanced ALS. As eye movements often represent the final channel of patient expression, their gradual deterioration destabilizes decision-making, complicates the interpretation of advance directives, and challenges the alignment of ongoing treatment with patient preferences. While communication difficulties are one of the most frequently reported concerns among patients and families following tracheostomy ventilation, phases of communicative uncertainty are largely absent from existing quality-of-life literature in ALS [46].

**Fig. 1:**
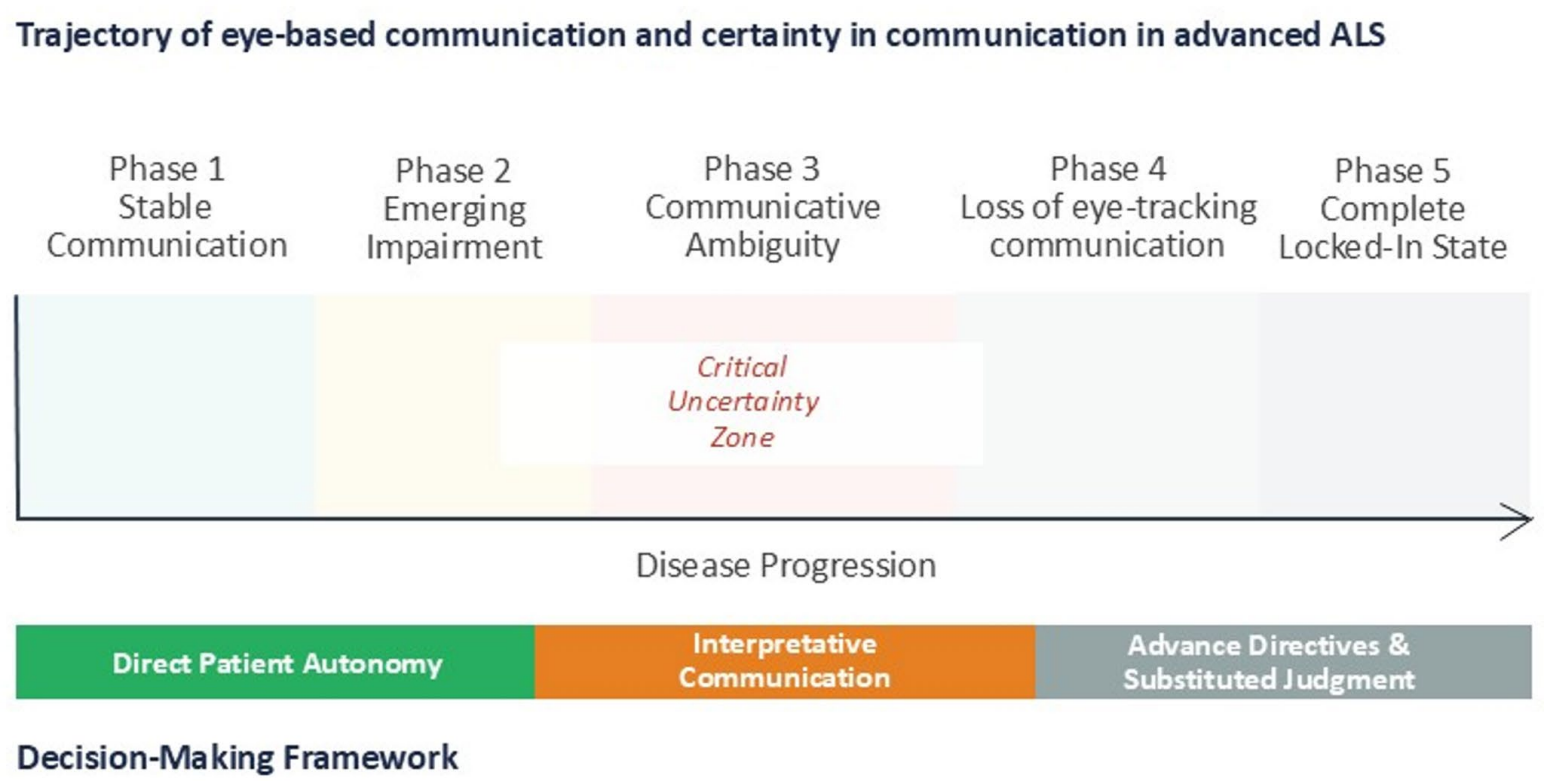
Trajectory of eye-based communication and certainty in advanced ALS. The figure illustrates the progressive decline of communication in ventilated ALS, from reliable eye-tracking, and therefore stable communication, to a complete locked-in state. The critical phase of uncertainty is characterized by inconsistent and difficult-to-interpret signals and marks a key challenge for clinical decision-making. The lower panel depicts the corresponding shift from direct patient autonomy to reliance on advance directives and substituted judgment

As ETCS failure is typically gradual rather than abrupt, patients, families, and care teams experience a phase of increasing effort, error frequency, frustration and fatigue. This trajectory is conceptually illustrated in Fig. 1. In the phase of communicative ambiguity, residual but unreliable eye movements may still be interpreted as meaningful communication, even when the patient’s ability to intentionally generate signals is already severely compromised. We therefore define communicative ambiguity as a transitional state in which observable communicative signals persist but can no longer be reliably attributed to intentional, patient-controlled expression. This state of communicative ambiguity creates ethical tension in ALS care, as observable signals continue to influence clinical decisions despite their uncertain intentional status and can further be complicated by cognitive changes that can limit the ability to make decisions [47]. Due to the given communicative ambiguity, the trajectory and form of these potential cognitive changes can no longer be diagnosed with certainty. This tension was reflected in Case 1, when residual ocular flickering was variably interpreted as intentional communication and will to live, and in Case 2, where inconsistent eye signals contributed to escalating uncertainty in goal-of-care decisions. Communicative ambiguity thus represents a critical threshold in ALS, marking the transition from direct patient autonomy to interpretative and surrogate-based decision-making.

Communication loss in ALS is not only a clinical transition but also a relational process. Families may experience the gradual loss of interaction as (anticipatory) grief, while healthcare professionals, and particularly nurses in intensive long-term care settings, may develop strong bonds that complicate their professional roles [48]. For healthcare professionals, communication impairments are a source of significant burden, as they hinder the ability to understand patients’ wishes, involve them in decision-making, and respect their autonomy [49].

Evidence highlights the significant emotional burden of end-of-life decision-making in ALS, particularly during ventilatory withdrawal. Both, nursing teams (as reflected in the presented cases) and clinicians, experience this process as highly distressing, time-consuming, emotionally intensive, and at times associated with long-term psychological impact [50]. Evidence from nursing perspectives further illustrates how communication decline directly impacts decision-making in clinical practice [48] and contributes to ethical ambiguity in situations where patients’ intentions and preferences cannot be reliably assessed [51]. Although non-verbal and empathic forms of interaction can help maintain relational connection, they often lack the precision required for complex medical decisions [48]. Moral distress can arise when previously documented patient preferences conflict with caregivers’ reluctance to accept the transition toward end-of-life care. Thus, the ethical challenge is not limited to respecting individual autonomy but involves navigating a complex interpersonal process where the patient’s will is interpreted under heavy emotional pressure. These ethical tensions are intensified by differing relational perspectives within care, as illustrated in Case 1, where family members interpreted ongoing treatment as prolonging suffering, whereas nursing staff perceived residual signals as indicating a will to live.

Trust between healthcare professionals and families can facilitate shared decision-making, whereas its absence, particularly in situations of limited communication, may lead to heightened control by caregivers, emotional strain, and barriers to person-centered care. In such contexts, communication impairment not only limits patient autonomy but also reshapes decision-making dynamics.

In the context of communicative ambiguity and absent or insufficient ACP, decision-making is frequently “defaulted” to relatives or clinicians, which can increase the risk of conflict. If decisions are shifted to relatives or clinicians, these are typically based on prior conversations, general attitudes, or inferred preferences rather than explicit directives [45]. This highlights the ethical fragility of substituted judgment in the context of advanced ALS and impaired communication, even when these are documented in advance directives by proxy [40]. This shift introduces significant ethical fragility and can cause moral distress when caregivers must navigate the conflict between a patient’s previously documented wishes and the emotional pressure of interpreting uncertain, late-stage signals.

#### Clinical implications for practice

Based on these experiences, ACP in patients with ALS and TIV must be reframed from a discrete event into an iterative and preemptive process specifically attuned to oculomotor decline. Patients with rapid progression of the disease are especially at risk of developing ophthalmoparesis, as an early need for TIV and becoming totally quadriplegic within 24 months of ALS onset seem to be predictors of severe communication impairment [20].

Framing communication decline as due to disease progression can help families and care teams contextualize uncertainty and moral distress, reducing the risk of attributing responsibility to caregivers or technology. For the clinical team, recognizing the first signs of ETCS decline should serve as a mandatory trigger for re-evaluating goals of care.

However, in clinical practice, ALS patients receiving TIV are frequently managed in home care settings and may no longer be regularly seen by neurologists [35]. This creates a structural discontinuity in care and increases the risk of missing the critical window for anticipatory ACP, before the onset of communicative ambiguity. Close and continuous collaboration between neurologists and palliative care teams is essential, both to ensure that goals-of-care discussions take place while communication remains sufficiently reliable and to provide consistent support during the end-of-life phase. The importance of understanding patients’ and caregivers’ perspectives on palliative care needs is increasingly recognised and is currently being investigated in a nationwide multicentre study in Germany [52].

**Fig. 2:**
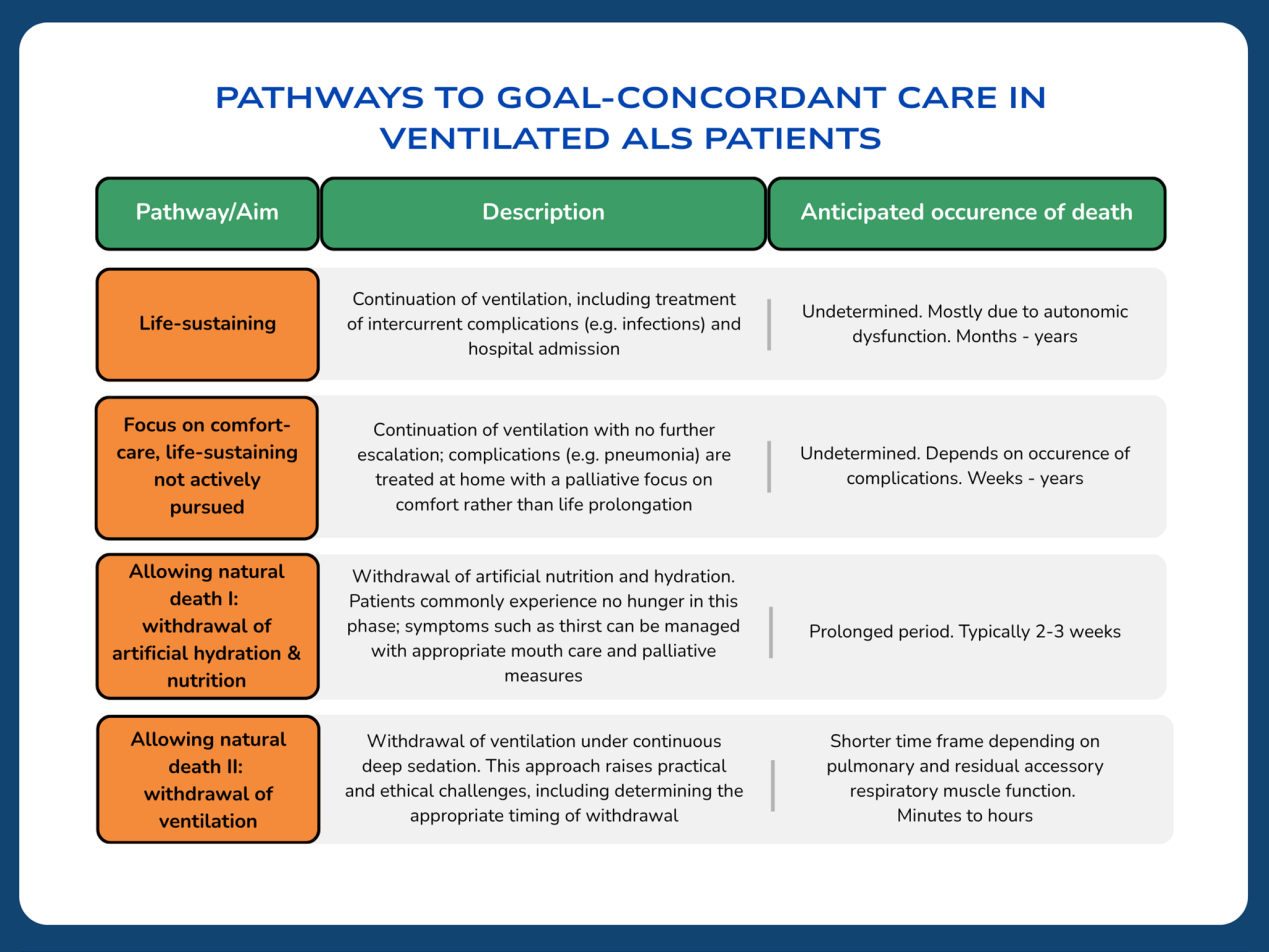
Pathways to goal-concordant care in ventilated ALS patients. Approaches differ in treatment goals, management strategies, and anticipated time to death, with important implications for decision-making as communication abilities decline

From a clinical perspective, we propose four principal pathways once a transition in treatment goals is considered in TIV ALS patients (*see* Fig. 2). These pathways represent a pragmatic framework that has evolved from our clinical experience in the neuropalliative care of patients with ALS and in the care of long-term ventilated patients with severe acquired brain injury; they are not derived from an established conceptual model. These options need to be carefully aligned with the patient’s preferences, values, and previously expressed wishes. Determining the most appropriate pathway and timing requires an individualized, balanced approach that also considers the patient’s communicative abilities, symptom burden, psychosocial context, and the perspectives of family and care teams. At the same time, decisions remain dynamic and may need to be revisited and revised in response to clinical changes. Accordingly, decision-making in this phase is less about selecting the best option than about continuously negotiating a trajectory of care that remains consistent with the patient’s goals in the face of advancing disease and increasing uncertainty.

Structured professional support for healthcare professionals is essential. This need is underscored by evidence demonstrating the profound emotional burden associated with end-of-life decision-making in ALS, particularly during ventilatory withdrawal, which clinicians experience as highly distressing, time consuming, emotionally intense, and sometimes associated with lasting psychological effects [49, 53]. Clinical ethics consultations, interprofessional case discussions, psychosocial and spiritual support can facilitate interpretation of uncertain signals, support decision-making based on previously expressed wishes, and help alleviate emotional burden.

## Conclusion

Progressive oculomotor dysfunction in ALS is a clinically significant marker of disease progression, with implications for communication and decision-making. However, beyond the loss of motor function, the gradual deterioration of eye-based communication introduces a clinically critical transition period (*see* Fig. 1). As eye-tracking communication often constitutes the final available channel for interaction, its progressive instability creates a phase of increasing uncertainty in interpreting patient intent. This has important consequences for clinical decision-making and ACP, as patient autonomy becomes progressively more difficult to assess. Communicative ambiguity represents a critical yet underrecognized phase in ALS, during which the interpretability of patient intent becomes fundamentally unstable. Failure to recognize this phase risks misattribution of communicative signals and may undermine the ethical validity of decision-making.

Proactive and iterative ACP is essential, not only to document patient preferences early, but also to anticipate the potential loss of reliable communication. Interdisciplinary collaboration, including neurology, palliative care, and psychological support, is important to ensure that decision-making remains aligned with previously expressed values when direct communication is no longer possible.

Importantly, the findings of this review highlight that communication loss in ALS is not a discrete event but a gradual process that may include a clinically relevant period of communicative ambiguity. Recognizing and addressing this transition is essential to reduce the risk of misinterpretation, support families and healthcare professionals, and ensure ethically sound decision-making in advanced disease.

## Data Availability

No datasets were generated or analysed during the current study.

## Abbreviations

ALS: Amyotrophic Lateral Sclerosis
TIV: tracheostomy-invasive ventilation
ETCS: eye-tracking communication systems
ACP: Advance Care Planning
PEG: percutaneous endoscopic gastrostomy
QoL: Quality of Life
